# Spontaneous Regression after Extensive Recurrence of a Pediatric Cervical Spine Aneurysmal Bone Cyst

**DOI:** 10.1155/2014/291674

**Published:** 2014-01-09

**Authors:** Carlo Brembilla, Luigi Andrea Lanterna, Michela Bosisio, Paolo Gritti, Andrea Risso, Antonio Signorelli, Francesco Biroli

**Affiliations:** ^1^Department of Neurosurgery, Pope John XXIII Hospital, OMS Square No. 1, 24100 Bergamo, Italy; ^2^Department of Pediatrics, Pope John XXIII Hospital, OMS Square No. 1, 24100 Bergamo, Italy; ^3^Department of Anaesthesia and Intensive Care, Pope John XXIII Hospital, OMS Square No. 1, 24100 Bergamo, Italy

## Abstract

Aneurysmal bone cyst is a pseudotumoral lesion. Complete resection prior to selective arterial embolization seems to be the treatment of choice for the more extensive and destructive lesions. In these cases maintaining stability of the cervical spine is critical. This can be very challenging in children and adolescents in whom the axial skeleton is still growing. In this case a young girl presented with a voluminous cervical aneurysmal bone cyst encaging both vertebral arteries and spinal cord. The lesion was treated with aggressive surgical resection, followed by cervical vertebral fusion with instrumentation. After nine months the patient referred no pain and no neurological deficit. MRI scans showed an extensive local recurrence. The family of the young girl refused any other therapy and any other followup. The patients returned to our attention after five years with no pain and neurological deficit. Cervical spine radiographs and MRI scans showed a complete regression of the extensive local recurrence. In the literature, the possibility of spontaneous regression of residual part or local recurrence is reported. The case of this young girl provided the chance to attend a spontaneous regression in an extensive recurrence of aneurismal bone cyst.

## 1. Introduction

Aneurysmal bone cysts are benign, highly vascular lesions characterized by expansible blood-filled cystic cavities [1–3]. First described by Jaffe [4] and Lichtenstein [5] in 1942, these lesions constitute 1.4% of all primary bone tumours and 15% of all primary spine tumours [6, 7]. They most often affect children and adolescents, with a slight female preponderance [7]. They are responsive to different treatments and in rare instances can mature and resolve, but local recurrences are described with any type of management. The diagnosis is not always easy and their potential for rapid growth, considerable destruction of bone, and neurological involvement has led to aggressive therapy [8–10]. Complete resection prior to selective arterial embolization with or without reconstruction and stabilization seems to be the treatment of choice for extensive and destructive lesions. In these cases maintaining stability of the cervical spine is critical. This can be very challenging in children and adolescents in whom the axial skeleton is still growing [11, 12]. Special interest must be paid to the possibility of spontaneous regression and resolution after incomplete removal, particularly in recurrent cases, with attention to avoid more aggressive treatment than necessary [13, 14].

## 2. Case Illustration

A 13 year-old female with a 2-month history of gradually worsening cervical pain was referred to this institution. She reported progressive weakness on left arm and progressive growth of a mass in left supraclavicular fossa (*Ø* ≈ 5 cm). On admission, physical examination revealed a grade 4/5 muscle strength in internal and external rotation of the shoulder, elbow flexion, and wrist extension on left arm. Left brachioradialis reflex was hypovalid. Hypoesthesia at the distribution of the fifth and sixth left cervical nerve roots was present. There was a positive Modified Spurling's Maneuver on left side.

**Figure 1:**
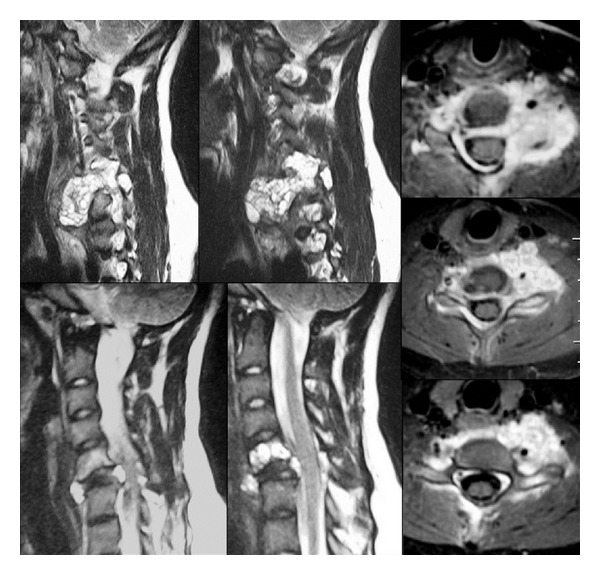
MRI scans of the cervical spine, showing a “soap-bubble” lesion of C5 and C6 with anterolateral paraspinal extension, which encaged both vertebral arteries and spinal cord.

Plain radiographs of the cervical spine showed an osteolytic lesion of C5 and a complete collapse of C6 vertebral body. Computerized tomography confirmed the standard radiological findings of the cervical spine, showing a ballooning, multilocular lytic lesion with a blown-out thin layer of cortical bone instead of C5 and C6 vertebral body. Magnetic resonance (Figure 1) showed a “soap-bubble” appearance of the lesion. The lesion had an anterolateral paraspinal extension, larger on left side, with encagement of both vertebral arteries. The mass also involved epidural space, in its anterior and posterior part, causing neural compression. The intervertebral discs were intact and not involved by the lesion.

An open biopsy performed according to oncologic principles revealed preponderance of fibrous tissue made of fibroblast, myofibroblast, and hemosiderin deposits, with enlarging vascular spaces and multinucleated osteoclast-like giant cells with some mitosis. A preoperative angiogram demonstrated stenosis and a tumoral blush of the left vertebral artery. The patient tolerated left vertebral artery occlusion test.

On the basis of all these findings anterior and posterior surgical resection with reconstruction and internal stabilization was felt to be the optimal approach. In order to permit a lesion removal as large as possible, left vertebral artery was embolized. First an anterior cervical approach was performed, with intralesional excision. Left vertebral artery was sacrificed. Not all the marginal limits of the lesion were included in the resection. A little residual part of the disease was left around right vertebral artery in order to avoid damage on it. An anterior spinal stabilization was performed using a titanium mesh packed with autogenous bone graft from the iliac crest and C4–C7 anterior cervical plating. After the first operation posterior cervical approach was performed with decompressive laminectomy and posterior spinal stabilization of C3–D1.

The histological response of the lesion was aneurysmal bone cyst. The patient's postoperative course was characterized by transient dysphagia, transient dysphonia, and stability of muscles strength. The patient walked with a cervical brace for three months and was referred to departments of physical medicine for proper rehabilitation therapy.

**Figure 2:**
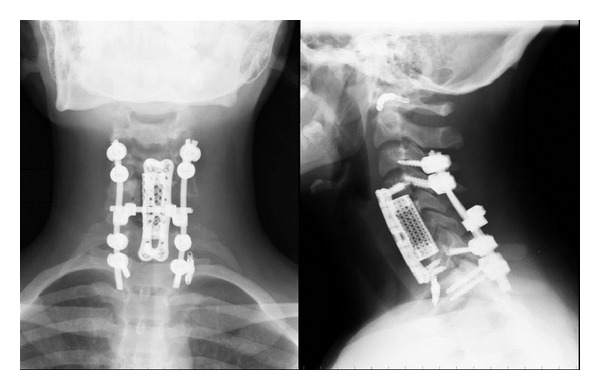
Postoperative cervical spine radiographs.

**Figure 3:**
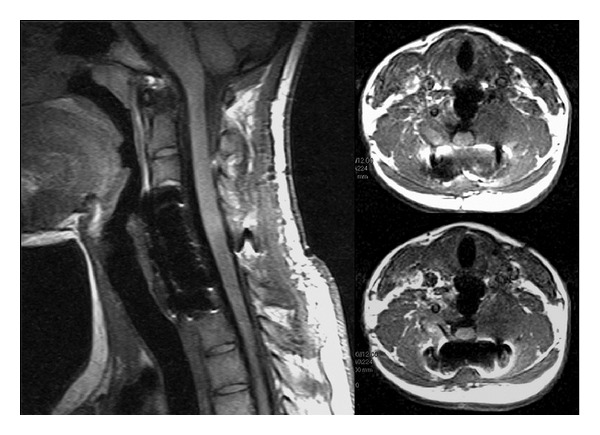
Six-month MRI scans, showing stability of the residual part of the lesion left around right vertebral artery.

**Figure 4:**
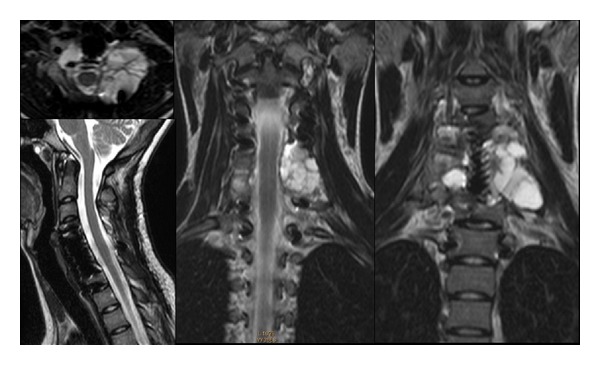
Nine-month MRI scans, showing an extensive bilateral recurrence more represented on the left side, with a characteristic “soap-bubble” appearance.

On postoperative day 1, two plain radiographic scans were performed (Figure 2). Two plain cervical radiographic scans and flexion extension lateral radiographs were obtained at 6 weeks and 3 months, to assess grade of fusion and correct alignment of the levels treated. The 1 mm thin-slice CT scans were performed at 6 weeks and 6 months, and were necessary to assess bone healing and placement of the implants. MRI scans were performed at 3, 6, and 9 months for monitoring the residual tumor. Until 6 months MRI scans showed stability of the residual part of the lesion left around right vertebral artery (Figure 3). After 9 months MRI scans showed an extensive bilateral recurrence (Figure 4). The lesion was more represented on the left side with a characteristic “soap-bubble” appearance. The patient referred no pain and no neurologic deficit. The young girl and her family were informed about the necessity of additional therapy (embolization and possible surgical reoperation), the natural history of the pathology, the risk of neurological deficit, and vertebral column instability. They refused any other therapy under their responsibility, because of the good clinical status. A program of followup with CT and MRI scans was planned, but they never attended it.

**Figure 5:**
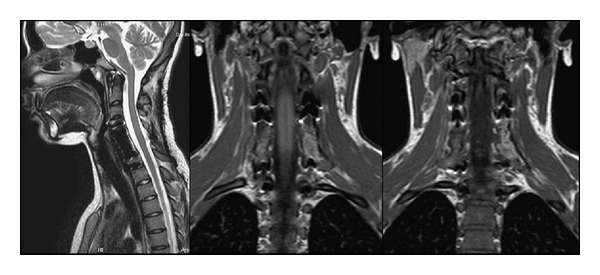
Five-year MRI scans, showing a complete regression of the extensive local recurrence.

The patient returned spontaneously to the attention of our institution after five years. She referred no pain and neurological deficit. Cervical spine radiographs showed a correct alignment of the levels treated, correct placement, and stability of the implant. MRI scans showed a complete regression of the extensive local recurrence (Figure 5). An annual followup with MRI scans was planned.

## 3. Discussion

Aneurysmal bone cyst (ABC) is a pseudotumoural hyperemic-hemorrhagic lesion of unknown etiology [1–3]. The lesion, first described by Jaffe [4] and Lichtenstein [5] in 1942, constitutes 1% to 6% of all primary bone tumours. The annual incidence of the disease is 0.14/10,000 per population [6, 7]. Female patients are slightly more affected. The majority of ABCs appear before the age of 20. The lesion is usually found in the femur, tibia, humerus, and spine [15, 16]. ABCs represent approximately 15% of all primary spine tumours, with 8% to 30% of these tumours arising from the mobile spine. The lumbar spine is most affected (40–45%), with lesions in the cervical (30%) and thoracic spine (25–30%) occurring less commonly. The posterior elements and the pedicles are usually affected first, and in 60% to 70% of cases the lesions extend into the vertebral body [17]. Progressive expansion and bone destruction of the vertebral body can ultimately lead to sudden collapse or angular deformity of the vertebral column and acute epidural compression of the spinal cord [16, 18]. In approximately 25% to 35% of cases, the lesion crosses over to an adjacent vertebra through surrounding soft tissues, but never through the intervertebral disc.

Common clinical signs are pain, often worse at night and with recumbency, and swelling; the duration of symptoms prior to diagnosis is usually 4 to 8 months. Rarely, acute vertebral collapse, with or without spinal cord injury, may be the presenting symptom [16].

The combination of standard radiographs, computed tomography (CT), and magnetic resonance imaging (MRI) provide useful information. The radiographic and CT scans typically reveal a characteristic soap-bubble appearance with fluid-fluid levels, which represents a ballooning, multilocular lytic lesion; this is pathognomonic in many cases [18].

Treatment options include simple curettage with or without bone grafting, complete excision, embolization, radiation therapy, or a combination of these modalities, but local recurrences are described with any type of management [19, 20]. The treatment of an ABC of the spine depends on the region of the spine in which it is located, the anatomic location of the tumor, and whether there is evidence of spinal cord compression or impairment of the structural integrity of the spine.

The aim of selective arterial embolization in the treatment of ABCs is to reduce the vascularity of the lesion, as well as to decrease intraoperative blood loss as a preoperative procedure [9, 10]. Many authors have also reported that percutaneous embolization is effective in the complete ablation of small and less destructive lesions [21–25]. Despite these encouraging reports, caution is required, because neovascularization may allow the embolic material to embolize outside the lesion.

Radiation therapy is limited for these lesions and sometimes associated with poor results [11, 14]. Radiation adversely affects growth in children with consequent late deformities. It may also cause late effects on the spinal cord itself as myelopathy and myelitis or severe radioinduced sarcoma [1, 15]. Radiation therapy should not be the first line of treatment for spinal aneurysmal bone cysts, and it remains an adjuvant therapy.

Although curettage and bone grafting have been reported to be successful in the management of aneurysmal bone cysts in the long bones of the extremities, the same does not apply to lesions of the spine [15, 16]. Complete resection, previous selective arterial embolization, is the treatment of choice with aneurysmal bone cysts of the spine [19, 26]. Excision must include the entire cyst wall because partial excision is related to a higher risk of recurrence. Curettage using a high-speed drill is often used to cut back to healthy, well-mineralized bone. If instability and/or deformity already exists, or if the amount of bone resection is expected to result in instability, simultaneous reconstruction and instrumented stabilization should be planned [9, 10, 20]. *En bloc* excision, if correctly planned and performed, is the only treatment option that has a 0% of local recurrence [11]. However this treatment option still remains surgically demanding because of intraoperative and postoperative high morbidity and does not seem justified by the clinical course of the disease. Special attention must be paid to the possibility of spontaneous regression and resolution after incomplete removal, particularly in recurrent cases, with attention to avoid more aggressive treatment than necessary [13, 14].

Spinal reconstruction is particularly important in pediatric patients. Extensive fusion and stabilization with instrumentation have often been avoided in children due to problems of future axial skeleton growth [27]. Despite the risk of postoperative deformity, in children it is usually recommended to use an orthesis until skeletal maturity before performing an arthrodesis [11].

In this case a young girl, presented with a voluminous cervical aneurysmal bone cyst encaging both vertebral arteries and spinal cord, associated with initial neurological symptoms. The osteolytic cervical lesion of C5 and C6 was treated with surgical resection, followed by cervical vertebral fusion and instrumentation. This course of treatment resulted in good removal of deeply involved tumor and successful reconstruction of the spine. After nine months the patient referred no pain and no neurological deficit. MRI scans showed an extensive local recurrence. The family of the young girl refused any other therapy and any other followup. The patient returned to our attention after five years with no pain and neurological deficit. Cervical spine radiographs and MRI scans showed a complete regression of the extensive local recurrence and stability of the implant. In the literature the tendency for spontaneous regression of residual part or local recurrence is reported [13, 14]. The case of this young girl and the decision of her family to refuse any other therapy and followup provided the chance to attend a spontaneous regression in an extensive recurrence of aneurismal bone cyst of the paediatric cervical spine after surgical management.
